# Adaptive RAO ensembled dichotomy technique for the accurate parameters extraction of solar PV system

**DOI:** 10.1038/s41598-024-63383-3

**Published:** 2024-06-05

**Authors:** P. Ashwini Kumari, C. H. Hussaian Basha, Fini Fathima, C. Dhanamjayulu, Hossam Kotb, Ali ELrashidi

**Affiliations:** 1 School of Electrical and Electronics Engineering, Reva University, Bangalore, India; 2 NITTE Meenakshi Institute of Technology (Autonomous), Bengaluru, India; 3 Mar Baselios Christian College of Engineering & Technology, Kuttikkanam, Kerala India; 4 School of Electrical Engineering, Vellore Institute of Technology, Vellore, India; 5 Department of Electrical Power and Machines, Faculty of Engineering, Alexandria University, Alexandria, 21544 Egypt; 6 Electrical Engineering Department, University of Business and Technology, Ar Rawdah, 23435 Jeddah, Saudi Arabia

**Keywords:** Adaptive Rao algorithm, Single diode, Double diode, Enhanced Rao algorithm, Three diode model, Dichotomy, Root mean square error, Parameter extraction, Energy science and technology, Engineering

## Abstract

The parameter extraction process for PV models poses a complex nonlinear and multi-model optimization challenge. Accurately estimating these parameters is crucial for optimizing the efficiency of PV systems. To address this, the paper introduces the Adaptive Rao Dichotomy Method (ARDM) which leverages the adaptive characteristics of the Rao algorithm and the Dichotomy Technique. ARDM is compared with the several recent optimization techniques, including the tuna swarm optimizer, African vulture’s optimizer, and teaching–learning-based optimizer. Statistical analyses and experimental results demonstrate the ARDM's superior performance in the parameter extraction for the various PV models, such as RTC France and PWP 201 polycrystalline, utilizing manufacturer-provided datasheets. Comparisons with competing techniques further underscore ARDM dominance. Simulation results highlight ARDM quick processing time, steady convergence, and consistently high accuracy in delivering optimal solutions.

## Introduction

The increasing population and urbanization demand highlight the necessity of utilizing energy sources effectively. Solar energy, with its abundant availability, now plays a crucial role in meeting these demands. This energy shows the upper hand over various other renewable energy sources^1^. Due to various advantages in terms of low maintenance, less noise, less infrastructure Requirements^2^. Developments in sustainable energy sources are gaining prominence due to increased fossil fuel consumption leading to high CO_2_ emissions to the atmosphere. Solar energy being most promising, renewable energy solution supports the future grid by pumping surplus power^3–5^. Despite increased PV growth, high initial cost, varying climatic changes and least energy conversion ratios hinder the usage of PV generation to some extent.

Researchers are putting continuous efforts to improve the performance of solar cells^6^. The primary requirement is to obtain a reliable and accurate method to estimate the parameter of PV cell that can describe the actual behavior under any climatic condition^7–9^. The Photovoltaic (PV) model's multi-model and nonlinear properties pose a challenging problem in parameter extraction. Moreover, due to the nature of this problem, algorithms employed for its solution are prone to being trapped in local optima. Nevertheless, accurate parameter estimation remains crucial given the significant influence these parameters exert on the performance of the PV system in terms of current and energy production^10^. Verification of PV cell performance is anticipated using *P–V* and* I–V* curves. Characterization of PV cell is done using single, double, or triple diode electrical equivalent approach. Every modeling approach has its own pros and cons. Burden lies with respect to estimation of parameters.

**Figure 1:**
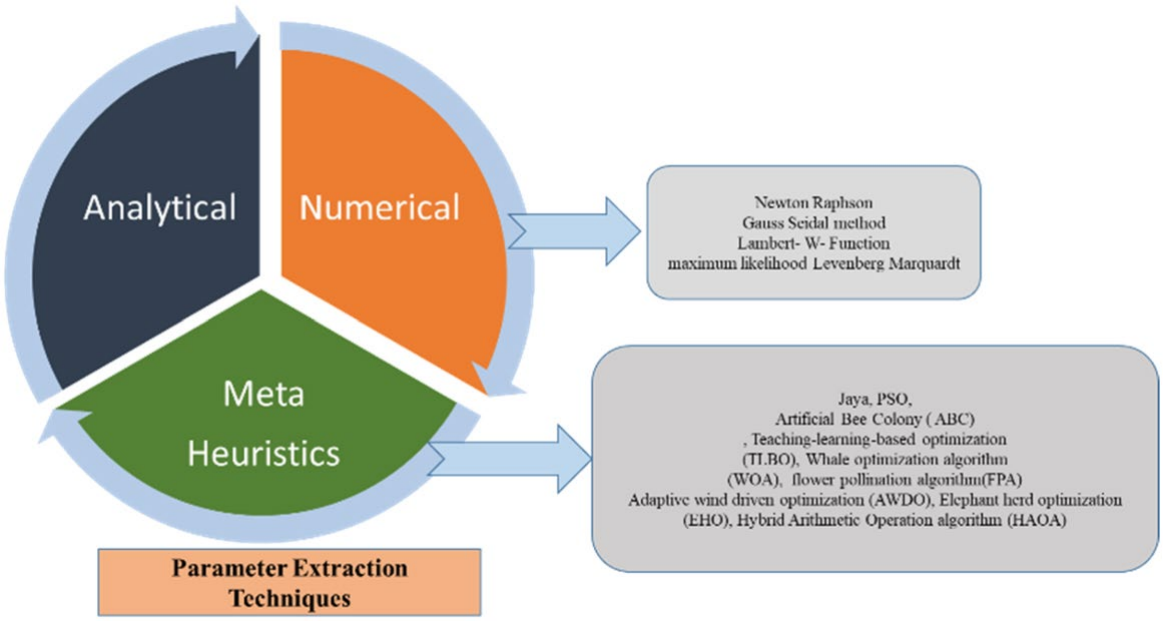
Different parameter extracting methods^21^.

Many of the application utilizes one diode model due to ease of implementation and fast estimation process with less complexity^11^. Several modeling approaches are presented in Fig. 1. Basically, it is categorized as analytical, numerical, metaheuristic and hybrid methods. Among these approaches metaheuristic is the best reported method in recent literature for extracting the unknown parameters. Five parameter model is also termed as single diode model and inclusion of additional diode generates a two-diode approach with good accuracy level by increasing the complexity of estimation to 7 parameters^12–15^. Imperfections accounted using one diode and double diode can be efficiently solved by using three diode model considering grain region leakage current of the PV Module. The data sheet obtained by the manufacturer describes major points on *P–V* & *I–V* curves at Standard test condition (STC) namely, open-circuit voltage, maximum-power, and current at short- circuit point. These three points provide mere knowledge to extract the parameters^16^.

Analytical methods differ from one study to another. To adopt this, the manufacture data sheet values are used at STC. Any slight changes in deviation will decline the accuracy of the estimated parameters^17^. Mathematical equations are derived based on approximation and by solving these equations analytical modeling is carried out by various authors. Nowadays curve fitting approaches are widely used due to their adequate and realistic accuracy^18^. Various approaches are extensively used for extraction of PV parameters. Numerical methods such as maximum likelihood Levenberg Marquardt, Newton Raphson, tabular and curve fitting approaches are also utilized with proper initial guests and step size. These methods require more computational time and can effectively address the problems associated with multidimensions and nonlinearity^19,20^.

Many stochastic methods are adopted in recent literature which mimic natural behavior such as swarm, bio inspiration, physics, and evolutionary techniques. Despite all these methods many times the solution is obtained by exploring and exploiting the search space, but the main drawback is some of these solutions get easily trapped into local minimum and the accuracy of the parameter estimator fails^22^. Most common algorithms include differential evolution (DE), cuckoo search, Jaya, PSO, ABC, TLBO, WOA, SCA, FPA, GCPSO (Guaranteed Convergence Particle Swarm Optimization) for estimation of unknown variables. Combining numerical and stochastic techniques addresses the main flaw in these numerical methods, improving accuracy and increasing system dependability^23^. These combinational methods help in exploiting and uncovering the wide solution range that can stop getting the trapped to local minima. Some of the widely used hybrid methods are published in literature^24^.

HISA is combined with interior search approach, a combination of the Newton–Raphson, levy-Flight, and Brownian-strategies, as well as the chaotic map and mutation schemes, are offered to reduce the number of iterations required for the optimization process^25^. The fluctuation in the performance of PV is damped by adjusting parameter using Levenberg Marquardt technique to estimate single and double diode parameters under different climatic condition. Adaptive wind driven optimization (AWDO) using practical and theoretical approach along with discussions on output current with experimental data are discussed in^26^. An improved version of modeling using general algebraic modeling is presented in^27^. This method includes linear, nonlinear, quadratic, and mixed integer optimization techniques. Single and two diode parameters are extracted using GAMS to verify the fake results produced by the researchers. Single, double, and triple diode models with 5, 7, and 9 variables are estimated using elephant herd optimization (EHO) and compared to improved performance based (EHO) variations such alpha tuning, culture based, and biased initialization technique.

Local minima are avoided by dividing the complete population into clan. Among the existing variant culture-based variants outperforms in different weather condition^28^. Slime mould approach with improvisation combined with Lambert W function is adopted to extend the investigation of two diode model during dynamic conditions^29^. Statistical Analysis of multipopulational trends and using statistical analysis, it is suggested that Rao be used to calculate the parameters of the one-diode and two-diode models. Self-adaptive optimization has no specific parameter with adaptation in population size. With respect to variation in population size, the gap between the old and new objective functions grows and shrinks with each iteration. However, these papers fail to estimate three diode approach which is very accurate. Enhanced Rao algorithm is presented in^30^ which includes repaired evolutionary operator and adaptive variation of population size. This evolutionary operator helps in escaping from the solution falling to local optimum.

## Modeling and approaches of solar PV cells

Three different models are used to demonstrate *I–V* and *P–V* performance characteristics namely one, two and three-diode approach.

### One diode model solar PV cell

**Figure 2:**
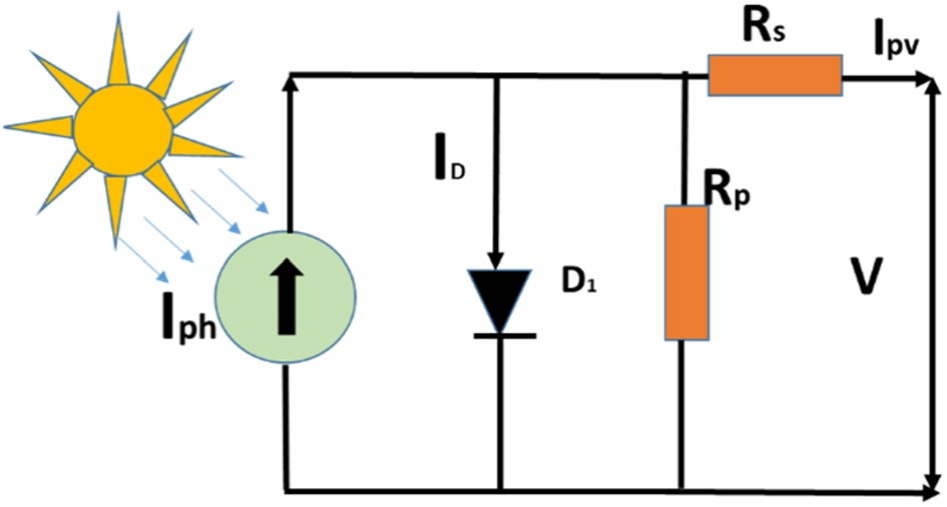
The equivalent circuit of single diode modeln size.

The schematic representation of the electrical equivalent schematic for a single diode model is depicted in Fig. 2., where five unknown PV parameters can be extracted. A one-diode model can be described by Shockley diode equation. Because it is easy to implement with only five parameters, it is commonly used for modelling solar cells^31^. The five parameters are (*I*_*ph*_, *I*_*d*_, *n*, *R*_sh_, *R*_s_). However, when the irradiation levels are low this model fails to describe the behavior of the cell accurately.

The current output is given by Eq. (1).

1 $${{\text{I}}_{{\text{pv}}}} = {{\text{I}}_{{\text{ph}}}} - {{\text{I}}_{\text{d}}}\left[ {\exp \left( {\frac{{{\text{V}} + {\text{I}} \cdot {{\text{R}}_{\text{s}}}}}{{{\text{n}} \cdot {{\text{V}}_{\text{t}}}}}} \right) - 1} \right] - \frac{{{\text{V}} + {\text{I}} \cdot {{\text{R}}_{\text{s}}}}}{{{{\text{R}}_{\text{p}}}}}$$

where *I*_*d*_ is the saturation-current, and *n* is the ideality factor, *R*_*s*_ and *R*_*p*_ are series and shunt resistance.

Where,

2 $${\text{V}}_{\text{t}}=\frac{{\text{N}}_{\text{s}} \cdot \text{K} \cdot \text{T}}{\text{q}}$$

### Two diode model solar PV cell

**Figure 3:**
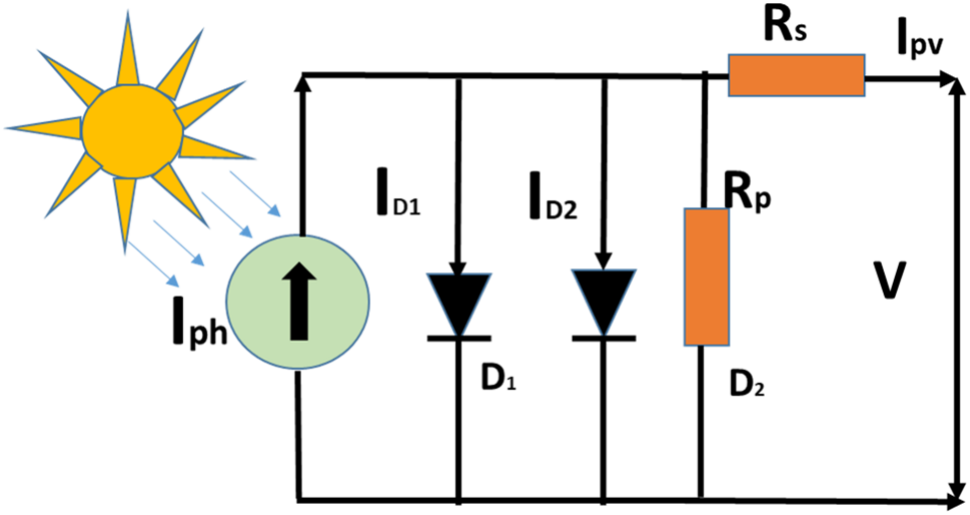
The schematic of double diode equivalent circuit modeln.

The model has 7 unknown parameters: I_ph_, R_p_, R_s_, Id_1_, Id_2_, n_1_, and n_2_. In parallel to the current source, an additional diode is added. Although this additional diode model achieves more precision than a single diode model, it necessitates more calculations due to its seven. Figure 3 depicts the double-diode model.

Here 7 parameters of the two-diode model are depicted in Eq. (3).

3 $${\text{I}}_{\text{pv}}={\text{I}}_{\text{ph}}-{\text{I}}_{\text{d}1}\left[{\exp}\left(\frac{\text{V}+\text{I} \cdot {\text{R}}_{\text{s}}}{{\text{n}}_{1} \cdot {\text{V}}_{\text{t}}}\right)-1\right]-{\text{I}}_{\text{d}2}\left[{\exp}\left(\frac{\text{V}+\text{I} \cdot {\text{R}}_{\text{s}}}{{\text{n}}_{2} \cdot {\text{V}}_{\text{t}}}\right)-1\right]-\frac{\text{V}+\text{I} \cdot {\text{R}}_{\text{s}}}{{\text{R}}_{\text{p}}}$$

Incorporating the extra diode includes recombination effect, resulting in better modeling.

### Three diode model solar PV cell

**Figure 4:**
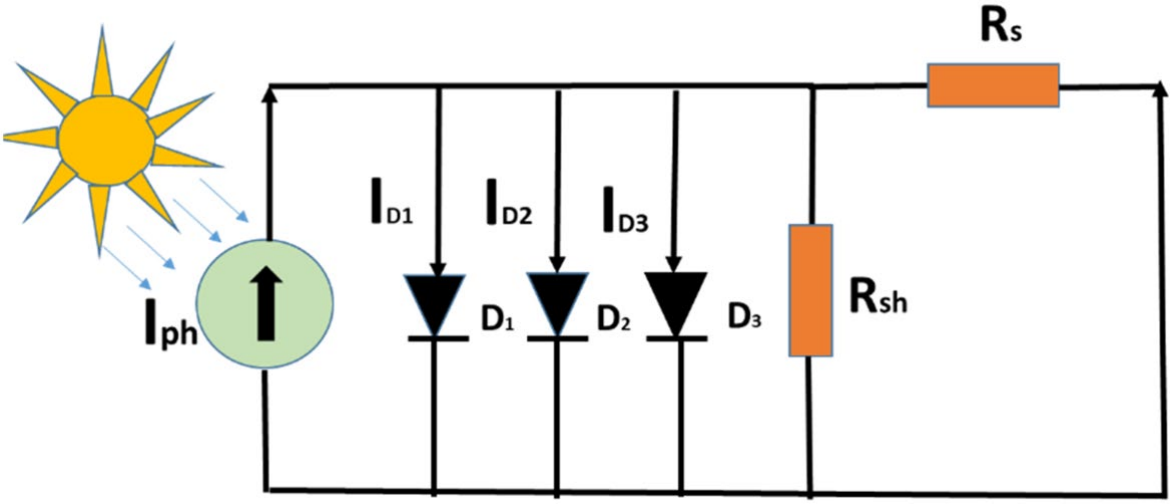
The equivalent circuit of three-diode approach.

In the TDM, the inclusion of a third diode allows for the representation of grain boundaries and leakage current, factors that were not adequately addressed in the SDM and DDM. This enhancement results in a more comprehensive model that better reflects real-world conditions and improves the accuracy of performance predictions for PV systems^32–39^. With a total of nine parameters, the TDM introduces greater complexity compared to its predecessors. However, this increased complexity is justified by the model's ability to provide more precise estimations of PV system behavior under varying operating conditions. Overall, the development of the TDM marks a significant step forward in PV modeling, offering researchers and engineers a valuable tool for optimizing system design and performance. The inclusion of an additional diode working in tandem with the existing two diodes in Fig. 3 enhances the two-diode method, resulting in the three-diode model. In contrast to the double-diode model, the three-diode model encompasses a total of ten parameters, specifically (*I*_*ph*_, *R*_*p*_, *R*_*S*_, *I*_*d1*_, *I*_*d2*_, *I*_*d3*_, *n*_*1*_, *n*_*2*_, *n*_*3*_, *K*). From the literature addition of third diode improves the accuracy of modeling and predicting the best performance characteristics. The three-diode model is represented as portrayed in Fig. 4.

The mathematical expression of the triple-diode approach is portrayed below.

4 $${\text{I}}_{\textrm{pv}}={\text{I}}_{\textrm{ph}}-{\text{I}}_{\textrm{d}1}\left[{\exp}\left(\frac{{\text{v}}_{\textrm{pv}}+{\text{I}}_{\textrm{pv}}{\text{R}}_{\textrm{s}}}{{\text{n}}_{1}{\textrm{V}}_{\text{t}}}\right)-1\right]{-\text{I}}_{\textrm{d}2}\left[{\exp}\left(\frac{{\text{v}}_{\textrm{pv}}+{\text{I}}_{\text{pv}}{\textrm{R}}_{\text{s}}}{{\textrm{n}}_{2}{\text{V}}_{\textrm{t}}}\right)-1\right]{-\text{I}}_{\textrm{d}3}\left[{\exp}\left(\frac{{\text{v}}_{\textrm{pv}}+{\text{iI}}_{\textrm{pv}}{\text{R}}_{\text{s}}}{{\textrm{an}}_{3}{\text{V}}_{\textrm{t}}}\right)-1\right]-\left(\frac{{\text{v}}_{\textrm{pv}}+{\text{I}}_{\textrm{pv}}{\text{R}}_{\textrm{s}}}{{\text{R}}_{\textrm{sh}}}\right)$$

Parameter identification of the given cell considers the set of *P–V* and *I–V* experimental data. The Fitness function is defined as either a minimization or a maximization function. The primary purpose of this publication is to minimize the discrepancy between the calculated data and the real data.

5 $$\text{f}\left({\text{v}}_{\textrm{pv}},{\text{i}}_{\textrm{pv}},\emptyset \right)={\text{I}}_{\textrm{ph}}-{\text{I}}_{\textrm{d}1}\left[{\exp}\left(\frac{{\text{v}}_{\text{pv}}+{\textrm{i}}_{\text{pv}}{\textrm{R}}_{\text{s}}}{{\textrm{n}}_{1}{\text{V}}_{\textrm{t}}}\right)-1\right]-{\text{I}}_{\textrm{d}2}\left[{\exp}\left(\frac{{\text{v}}_{\textrm{pv}}+{\text{i}}_{\textrm{pv}}{\text{R}}_{\textrm{s}}}{{\text{n}}_{2}{\text{V}}_{\textrm{t}}}\right)-1\right]-{\text{I}}_{\textrm{d}3}\left[{\exp}\left(\frac{{\text{v}}_{\textrm{pv}}+{\text{i}}_{\textrm{pv}}{\text{R}}_{\textrm{s}}}{{\text{n}}_{3}{\textrm{V}}_{\text{t}}}\right)-1\right]-\frac{{\text{v}}_{\textrm{pv}}+{\text{i}}_{\textrm{pv}}{\text{R}}_{\textrm{s}}}{{\text{R}}_{\textrm{sh}}}-{\text{i}}_{\textrm{pv}}$$

Fitness function is given by Eq. (4) which is the RMSE of the difference between the experimental and the computed data.

6 $$\text{Min }(\text{F}(\uptheta ))=\sqrt{\frac{1}{\text{N}}{{\sum }_{\text{i}=1}^{\text{N}}\left({\text{I}}_{\text{i}}-{\text{I}}_{\text{i},\text{ext}}(\uptheta )\right)}^{2}}$$

The objective function described in Eq. (4) can be classified as a transcendental equation. Numerous writers have posited the assumption of equivalence between the anticipated current and the actual one to circumvent this transcendental issue. By incorporating dichotomy to provide more accurate responses whenever the objective function is called, we have overcome this transcendental issue.

## Design of dichotomy method

At each iteration step in the dichotomy approach, the search interval for the solution is halved. The interval in which the solution is found is determined to get increasingly smaller^40^. The approach utilized in this study is grounded on the intermediate-value theorem. This theorem posits that if a function f(x) is continuous on the interval [a, b] and f(a) and f(b) have opposite signs, then there exists at least one real number c between a and b where f(x) is continuous and f(c) equals zero. This is since zero lies between the values of f(a) and f(b) on the interval [a, b]. Estimation of unknown parameters of PV model or cell employs heuristic method.

- The unidentified variables are calculated and replaced within the fitness function to verify the outcomes.
- The main objectives of this manuscript are to present the adaptive Rao algorithm with modifications.
- Inclusion of dichotomy method to the objective function, amalgamation of adaptation and dichotomy yields best RMSE compared to other methods existing in the recent research works.
- Three PV models namely one, two and three-diode approaches are used to estimate the parameters with unique Modification in algorithm to have a least RMSE.
- Benchmarking is done by contrasting the outcomes of the presented methodology with other methods that have been previously documented in the literature.

**Figure 5:**
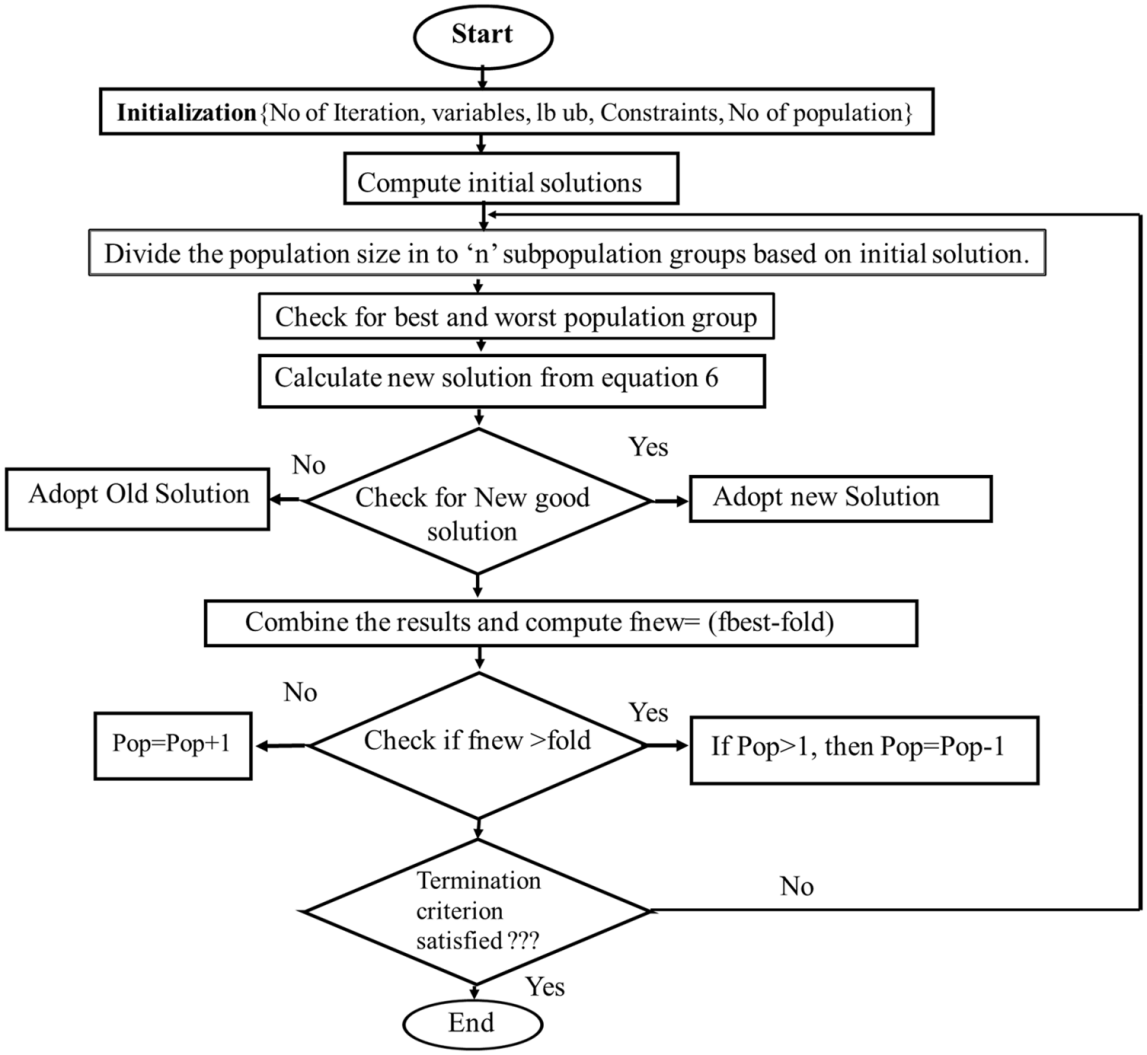
Flow chart of self adaptive multipopulational Rao algorithm.

The real time PV module is considered with experimental data and analysis to be performed to obtain least mismatch in the *P–V* & *I–V* curves. The Rao-algorithm is a straightforward optimization approach with no set parameters. The revised solutions in each iteration of the Rao algorithm are dependent on the best and worst solutions. This idea is presented in Eq. (6). Self-adaptive population Rao (SAP-Rao)^41^ is based entirely on the Rao algorithm. Based on the level of quality of the responses, this method splits the whole population into several groups, resulting in several subpopulations^42^. Instead of concentrating on a single area, the utilization of the number of sub-populations disperses the solutions across the search field. As a result, it is anticipated that the proposed algorithms will produce the best results. Each iteration of the SAP-Rao algorithm modifies the population size in accordance with the chosen objective function. As the gap between the previous and current target functions widened, so did the population size, and vice versa. As the gap between the previous and the current aim functions shrank, so did the population size. A flowchart for SAP-Rao is shown in Fig. 5.

7 $$ {\text{X}}\__{{{\text{new}}}} = {\text{ X}}\__{{{\text{old}}}} + {\text{ r}}_{{1}} \left( {{\text{X}}_{{{\text{best}}}} {-}{\text{ X}}_{{{\text{worst}}}} } \right) $$

## Discussion on results

### Case study 1: DDM of RTC—France PV cell

In order to assess the efficacy of ARD modifications in double and triple diode models, a set of 57 mm diameter commercialized solar cells made of silicon sourced from the RTC France cell. The experiment is conducted at 33 °C with irradiation of (1000 W/m^2^). The data sheet containing all the specification for RTC and Photo watt PWP (201) model is given in the Table 1.

**Table 1:** Datasheet of RTC France cell and PWP Photo watt (201).

Data sheet parameters	Values	Values
Voc (V)	0.5728	16.778
Isc (A)	0.7603	1.0300
Imp (A)	0.6894	0.9120
Vmp (V)	0.450	12.649
ki for Isc	0.0350%/C	–
N	1	36

**Figure 6:**
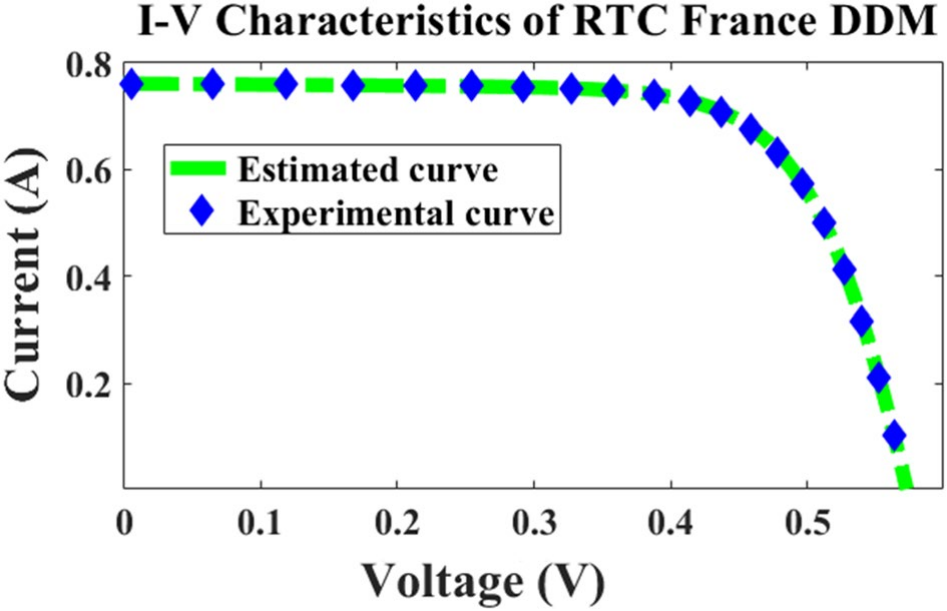
IV characteristic of RTC cell.

**Figure 7:**
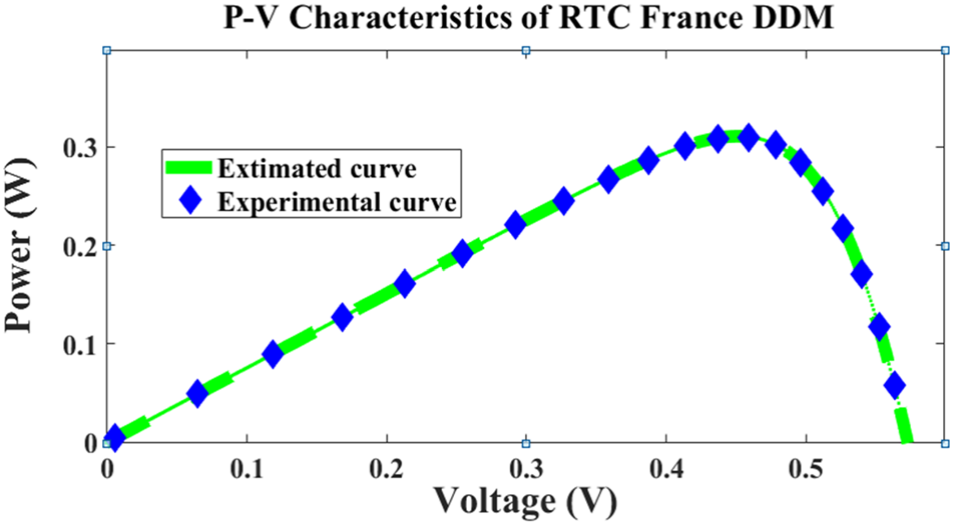
*P–V* characteristic of RTC cell.

**Figure 8:**
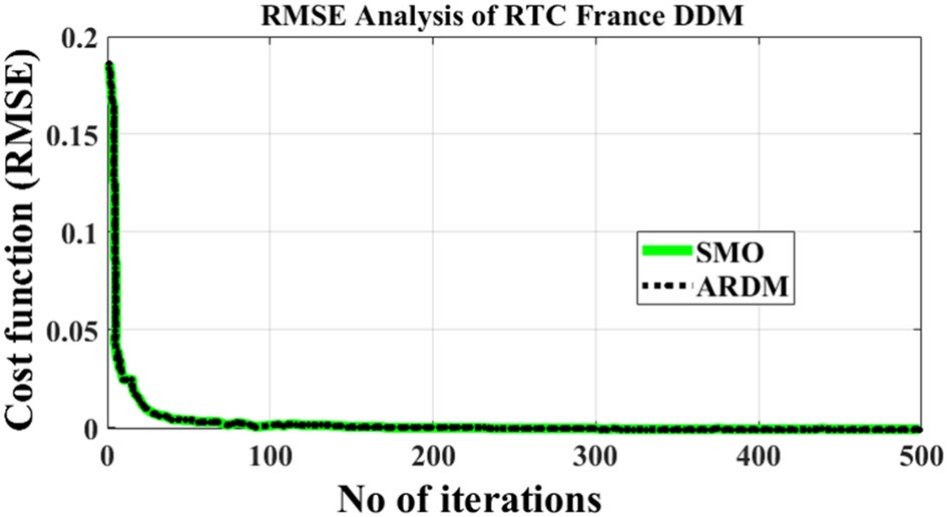
Convergence curves depicting the RMSE of fast performing method.

The computer simulation results proved that the proposed method outperforms better compared to other methods presented in the literature as demonstrated in Tables 2 and 3. The RMSE was found to be 7.3255 × 10^–4^ for double diode model with 7 parameters estimated and also 10 parameters were estimated with good accuracy of 7.3255 × 10^–4^ respectively, which is least in the comparison table presented in Table 4. Figures 6 and 7 illustrate the current–voltage *I–V* and power-voltage *P–V* characteristics of the RTC-France cell, showcasing both calculated and practical data. From the characteristics curve it’s clear that the error between these two values is very less illustrating better convergence as presented in Fig. 8. Convergence curves portrays that the number of iterations taken by the ARDM with respect to slime mould optimization techniques are quite similar, but the lower RMSE is achieved within 100 iterations which proves the accuracy of estimation with faster convergence speed.

**Table 2:** Double diode parameter extraction and comparison using different algorithms.

Parameter extraction methods	Parameters							
	I_ph_ (A)	I_o1_ (µA)	I_o2_ (µA)	a	a_2_	R_s_ (Ω)	R_p_ (Ω)	Best RMSE × 10^–4^
ARDM	0.7608	2.1772	0.08695	2	1.3712	0.0380	58.3713	7.3255
ELPSO^43^	0.76080	1e-6	0.099168	1.835767	1.386091	0.037551	55.920471	7.4240
C-HCLPSO^44^	0.76081	0.008797	0.97376	1.3795	1.817	0.37640	55.796	7.4259
MPSO^45^	0.760812	0.008971	2.136189	1.373644	2	0.037994	58.24134	7.3257
ISCE^46^	0.760781	0.225974	0.749348	1.451016	2.0000	0.036740	55.485444	9.824849
EHA-NMS^47^	0.760781	0.225974	0.749346	1.451017	2.000000	0.036740	55.485441	9.824848
BHCS^48^	0.76078	0.74935	0.22597	2.00000	1.45102	0.03674	55.48544	9.82485
PGJAYA^49^	0.7608	0.21031	0.88534	1.4450	2.0000	0.0368	55.8135	9.8263
DE-WAO^50^	0.760781	0.225974	0.749346	1.451017	2.00000	0.036740	55.485437	9.82484

**Table 3:** Comparison of metrics parameter for proposed and SMO for different iterations.

No of iterations	RMSE [ARDM]	RMSE [SMO]	Population size	Time in s
50	1.263 × 10^–03^	1.3719 × 10^–03^	20	6
150	1.0583 × 10^–04^	1.2150 × 10^–04^	40	19
250	9.86350 × 10^–04^	9.96084 × 10^–04^	60	27
500	7.3255 × 10^–04^	9.98034 × 10^–04^	100	34

**Table 4:** Comparison of RTC 3D model with Existing literature.

Methods	I_ph_ (A)	I_o1_ (µA)	I_o2_ (µA)	I_o3_ (µA)	n_1_	n_2_	n_3_	R_s_ (Ω)	R_p_ (Ω)	Best RMSE × 10^–4^
Proposed method	0.76072	2.1772	6.22E−06	0.00869	1.9999	1.3711	1.3712	0.03803	58.3713	7.3255
GBO^51^	0.7607769	0.78129	0.221556	0.00721	1.99	1.449	1.9756	0.036758	55.62330625	9.8250
SLTBO^52^	0.7608	0.2349	0.2297	0.4443	1.445	2	2	0.0367	55.2641	9.8253
CSA^53^	0.7603588	0	0.15567	0.2912	1.82	2	1.4717	0.036699785	58.93317	10.37665
OBWOA^54^	0.76077	0.2353	0.2213	0.4573	1.454	2	2	0.03668	55.4448	9.8249
MFO^55^	7.16E−01	0.42389	0.08147	0.01843	1.65	1.3909	1.4666	0.03713153	53.31146	9.97027

The brief comparison of SMO approach with reference to change in iteration and population size is tabulated in Table 3. From the table below it is clearly evident that with increased iteration and population size the RMS gradually decreases, but the taken to achieve least objective function increases. Thus, there is always trade-off between computational time and no of iterations.

### Case Study 2: photo watt PWP201 PV module SD

In order to determine the accuracy of the presented approach the commercial cell named photo watt PWP201 is considered with an objective of estimating five parameters of single diode model. It consists of 36 series-connected cells. Temperature is 45 °C with a 1000 W/m^2^ irradiance. The RMSE obtained by proposed approach is compared with that of the recent algorithms the RMSE was found to be 1.54 × 10^–3^ which is least among the various methods presented in Table 5.

**Table 5:** Comparison of photo-watt (PWP-201) with different algorithms.

Different methods	Parameters					
	I_ph_ (A)	I_o_ (µA)	n	R_s_ (Ω)	R_p_ (Ω)	RMSE × 10^–3^
ARDM	1.0301	1.18603	45.3986	1.51002	680.9921	1.540911
MPSO^56^	1.032230	2.552134	1.31884	1.238450	762.9058	2.041
WDO-WOA-PSO^57^	1.032382	2.512911	1.317304	1.239288	744.71435	2.046535
GCPSO^58^	1.032382	2.512922	1.317305	1.239288	744.71663	2.046535
TVACPSO^59^	1.031435	2.6386	1.321018	1.235611	821.59514	2.0530
SDA^60^	1.030517	3.481614	1.349970	1.201288	981.59961	2.425074
EHA-NMS^61^	1.030514	3.482263	1.35119	1.201271	981.98225	2.425075

### Case study 3: single diode parameter evaluation of experimental data

**Figure 9:**
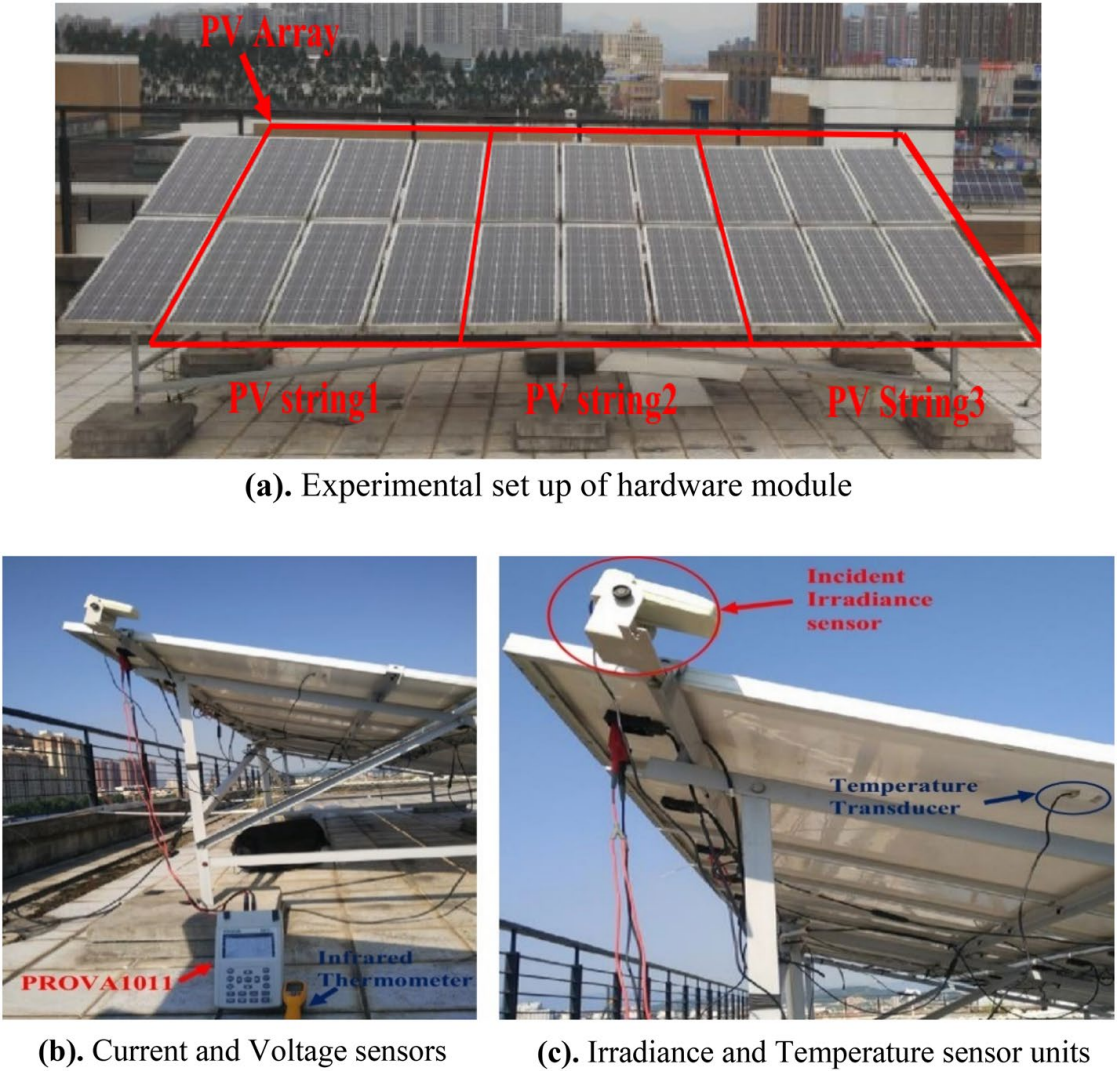
Experimental setup of (**a**) solar array, (**b**) current–voltage measuring sensors, and (**c**) irradiance and temperature sensor.

**Figure 10:**
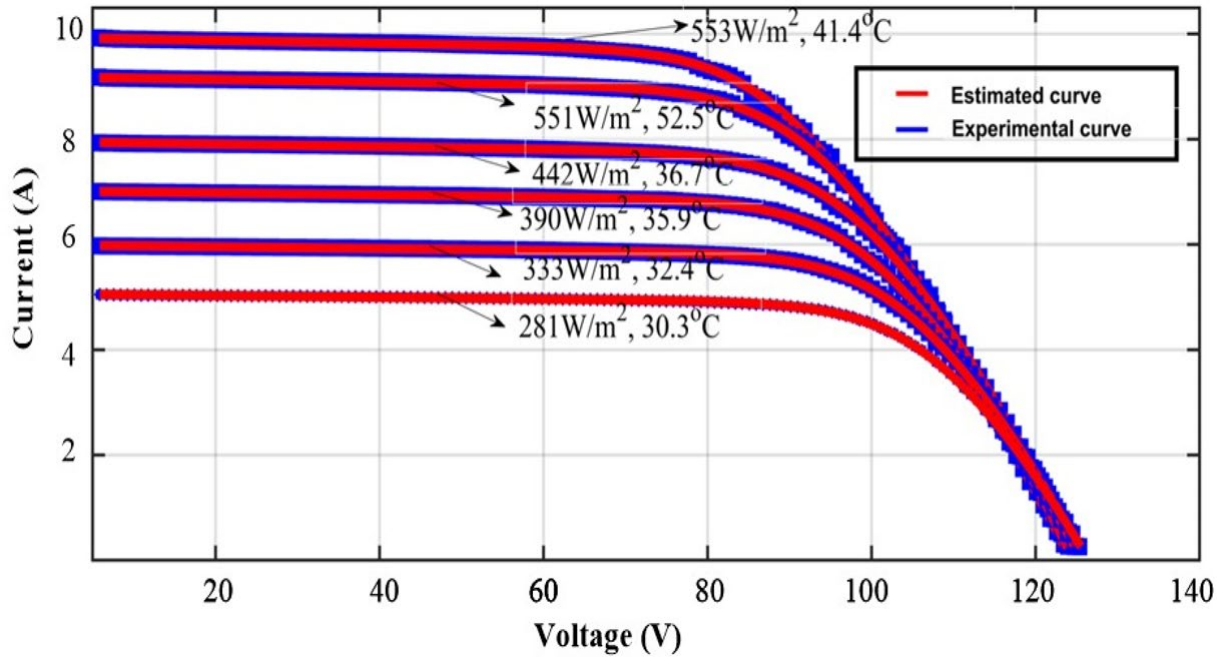
PV array (GL100) *I–V* characteristics at various temperatures.

The technique's performance was investigated using a large-scale system for implementation. The experimental results from the PV array in Fig. 9a–c are used in this case study. Three parallel strings with six PV modules each make up the used portion of the PV array. The monocrystalline GL-M100 PV module with 36 series-connected cells serves as the standard for PV modules. The PROVA1011, *I–V* testing instrument has been used to detect the *I-V* characteristics, including temperature and irradiance. The data sheet provided in the literature contains information on the PV module's electrical properties. The performance of ARD is demonstrated here using a single diode model. Six different temperature and irradiations were considered and for each case of irradiation and temperature 5 parameters of one-diode model is estimated and the RMSE is presented along with comparison with literature^62^ in Table 6. The *I–V* performance curves of PV array (GLM100) at various temperature are presented in Fig. 10. The curves show good match between the experimental and emulated values addressing the best accuracy.

**Table 6:** Real time analysis of effect of irradiation on parameter estimation.

Atmospheric conditions		Algorithms	Parameters estimated					Best RMSE
irradiance (W/m^2^)	Temperature (°C)		*I*_*ph*_ (A)	*Io* (µA)	*a*	*R*_*s*_ (Ω)	*R*_*sh*_ (Ω)	
553	41.4	AR-DM	10.0076	0.005976	5.99754	2.6917	360.6267	0.0254
		ABC-TRR^63^	10.00	0.0057	215.87	2.699	368.2	0.0580
511	52.5	AR-DM	9.2413	0.0098	6.1851	2.5401	412.1607	0.02213
		ABC-TRR^64^	8.00124	0.01276	6.46768	2.54568	426.17692	0.0580
442	36.7	AR-DM	8.0012	1.2604	6.3684	2.5506	426.4955	0.0166
		ABC-TRR^65^	8.00	0.0099	230.29	2.568	419.8	0.0313
390	35.9	AR-DM	7.0612	0.0036	6.0254	2.6665	487.4974	0.0128
		ABC-TRR^66^	7.06	0.0057	225.77	2.630	515.5	0.0291
333	32.4	AR-DM	6.0219	0.0024	6.1708	2.6608	561.7872	0.0097
		ABC-TRR^67^	6.02	0.0034	225.75	2.634	580.2	0.0182
281	30.3	AR-DM	5.0859	0.0022	6.2488	2.6531	603.3562	0.0069
		ABC-TRR^68^	5.08	0.0032	228.83	2.620	621.8	0.0134

The parameters of three diode model are also extracted for the proposed hardware and the analysis in variation of irradiation and temperature are done and tabulated in Table 7. By demonstrating that magnitude of RMSEs which is smaller than in the reference study^75^, this table supports the assertion that the ARDM algorithm accurately predicts the parameters when the PVs are subjected to a variety of temperature and irradiance conditions.

**Table 7:** Real time analysis of effect of irradiation and temperature on parameter estimation of three diode model.

	ARDM	SMO^69^	ARDM	SMO^70^	ARDM	SMO^71^	ARDM	SMO^72^	ARDM	SMO^73^	ARDM	SMO^74^
Parameters	G = 553; T = 41.4;		G = 551; T = 52.5;		G = 442; T = 36.7;		G = 390; T = 35.9		G = 333; T = 32.4		G = 281; T = 30.3	
*RMSE*	0.0579	0.058	0.0544	0.058	0.0313	0.0313	0.029	0.0291	0.0181	0.0182	0.013336	0.0134
*n1*	5.6105	5.996	9.0165	6.467	9.9724	230.29	9.151	225.7	6.2707	225.75	0.8798	228.8
*n2*	5.99638		2.2852		6.4739		2.686		6.2707		4.3884	
*n3*	6.64588		5.9775		6.11363		6.271		6.9747		0.3978	
*Iph*	10.0043	10.0007	9.23795	8.0012	7.99855	8	7.0551	7.06	6.01895	6.02	5.0813	5.08
*Rs*	2.69915	2.699	2.54461	2.5456	2.591345	2.568	2.6297	2.63	2.63445	2.634	2.6219	2.62
*Rp*	368.233	368.2	423.119	426.18	441.2868	419.8	515.45	515.5	580.2417	580.2	638.34	621.8
*Io1*	0	0.057E−06	0	0.0128	9.65E−07	0.0099	2.04004	0.0057	3.58E−10	0.0034	2.73E−68	0.0032
*Io2*	5.72E-09		0		0		0		3.03E−09		6.10E−31	
*Io3*	0		9.68E−09		3.60E−09		5.70E−09		0		3.65E−09	

Figure 10 displays the performance curves of the *P–V* for different operating conditions. The consistency between the empirical and estimated curves on the curves in Fig. 10 additionally demonstrates the precision of the results.

## Conclusion

In order to estimate the optimal values for the PV cell’s internal parameters, this article employed a novel Adaptive Rao algorithm in conjunction with the Dichotomy method (ARDM). The utilization of the dichotomy technique aimed to enhance the precision of the most effective estimating methods through the invocation of the goal function. The process was applied to a PV cell, a PV panel, and a PV array with 18 individual modules. The suggested ARDM methodology demonstrates a much-improved solution compared to the best existing techniques, as evidenced by the obtained findings and comparisons with other methods in the literature. This is indicated by the RMSE values of 1.54118 × 10^–3^ for the Photo-Watt PWP201 and 7.3255 × 10^–4^ for the RTC France PV module. The results of the experimental PV array with 18 panels shows that the algorithm is robust when panels are subjected to different environmental conditions, and the convergence curves that reach the optimum before the 500^th^ iteration demonstrate the speed of the algorithm. The effectiveness of a PV system relies on the optimal design of its cells and overall system, all of which would benefit from the approach suggested in this work. Faults in a PV system can be identified or predicted with the use of these parameters.

## Data Availability

The data used to support the findings of this study are included in the article.

## Abbreviations

PV: Photovoltaic
ODM: One diode model
TDM: Three diode model
ABC: Artificial bee colony
HISA: Hyperplanes intersection simulated annealing
LM: Levenberg Marquardt
NR: Newton Raphson
TSA: Tunicate swarm algorithm
AWDO: Adaptive wind driven optimization
GBO: Gradient-based optimize
OBWOA: Opposition-based whale optimization algorithm
GCPSO: Guaranteed convergence particle swarm optimization
FPA: Fower pollination algorithm
GAMS: General algebraic modelling systems
BHCS: Biogeography-based heterogeneous cuckoo search
SDA: Successive discretization algorithm
WOA: Whale optimization algorithm
DDM: Double diode model
DE: Differential evolution
RTC: Radio technique Comelec furnace
WOA: Modified three diode model
EHO: Elephant herd optimization
SCA: Sine cosine algorithm
HAO: Hybrid arithmetic operation
ARDM: Adaptive Rao with dichotomy method
MPSO: Modified particle swarm optimization
EHANMS: Hybrid adaptive Nelder–Mead simplex algorithm
TLBO: Teaching–learning-based optimization
ISCE: Improved shuffled complex evolution
EHA-NMS: Hybrid adaptive Nelder–Mead simplex algorithm based on eagle strategy
MFO: Moth flame optimizer
